# Endovascular repair of a type B aortic dissection with a right-sided aortic arch: case report

**DOI:** 10.1186/1749-8090-8-18

**Published:** 2013-01-23

**Authors:** Weimin Zhou

**Affiliations:** 1 Department of Vascular Surgery, the second affiliated hospital of Nanchang University, Nanchang, China

**Keywords:** Aortic dissection, Right-sided aortic arch, Stent-graft

## Abstract

Right-sided aortic arch is a rare anomaly, and aortic dissection involving a right-sided aortic arch is extremely rare. We report the case of a 65-year-old man with a right-sided aortic arch and a right descending aortic dissection and a stent-graft was accurately deployed without perioperative complications. There were no any complaints and complications after 18 months follow-up. The CTA demonstrated that the false lumen was largely thrombosed only with a mild type II endoleak and a mild descending aortic expansion. We feel that endovascular repair is feasible to patient of type B aortic dissection with a right-sided aortic arch. However, long-term clinical efficacy and safety have yet to be confirmed.

## Background

Right-sided aortic arch is a rare anomaly, and aortic dissection involving a right-sided aortic arch is extremely rare. To our knowledge, previous reports that we have identified in English literature include only fifteen cases of aortic dissection involving a right-sided aortic arch [1]*(through a Medline database search from 1966 to October 2011 and the search strategy “right-sided aortic arch and aortic dissection”)* and they were performed with traditional surgical procedures except for one report has described endovascular treatment [2]. We are presenting a case of type B aortic dissection associated with a right-sided aortic arch and aberrant left subclavian artery, in which a successful endovascular stent-graft placement was performed.

## Case presentation

**Figure 1:**
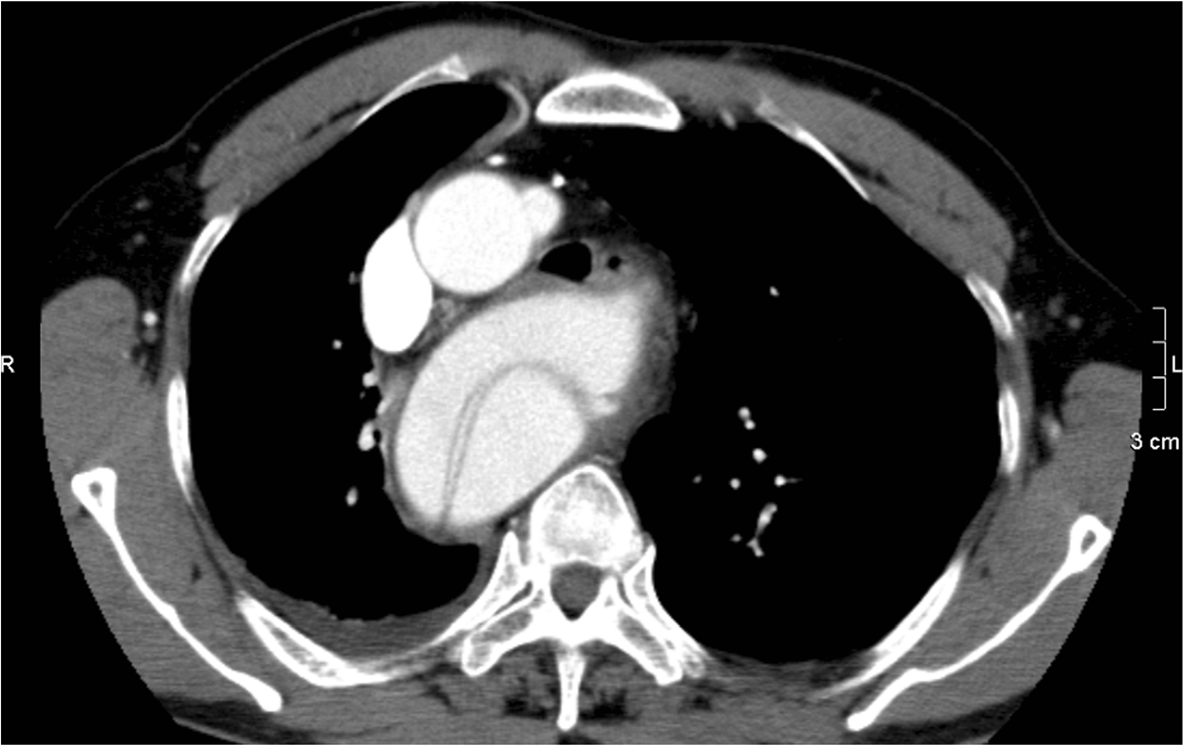
CTA demonstrated a right-sided aortic arch and a right descending thoracic aorta, a type B aortic.

A 65-year-old man was admitted to our institution with acute onset chest and back pain and computer tomographic angiography (CTA) demonstrated a right-sided aortic arch; a right descending aortic dissection with an aberrant left subclavian artery (LSA) arising from Kommerell’s diverticulum (Figure 1*). The size of descending aortic is 52mm.*It also defined the ordering of the aortic arch branches as left common carotid, right common carotid, right subclavian, and left subclavian arteries from proximal to distal; a celiac axis, superior mesenteric artery and right renal artery originating from true lumen and left renal artery originating from false lumen and a little pleural fluid in bilateralis thoracic cavity.

**Figure 2:**
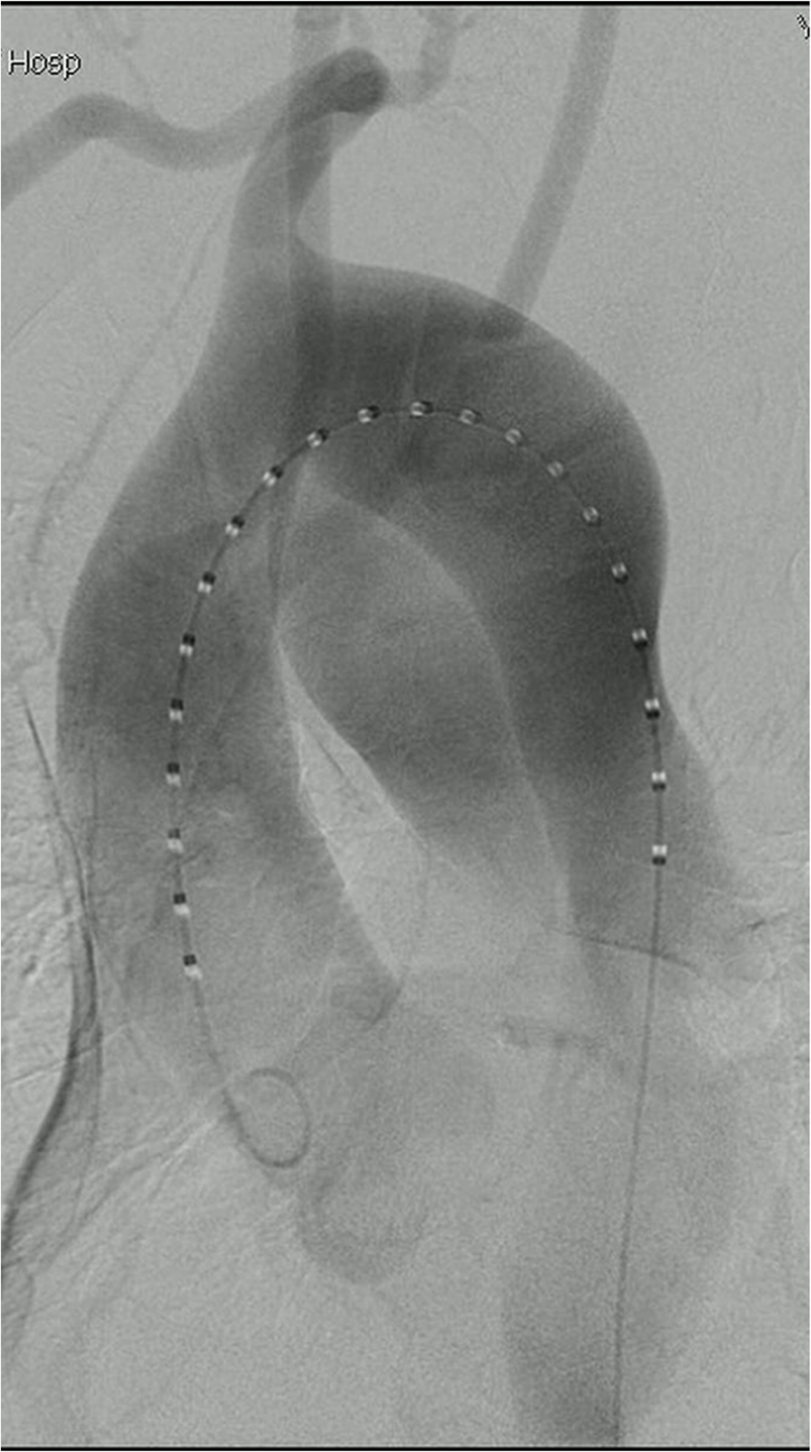
**Digital subtraction angiography showed a right-sided aortic arch and the right vertebral artery is dominant.** A LAO projection showed the ordering of the aortic arch branches as left common carotid, right common carotid, right subclavian and left subclavian arteries from proximal to distal.

**Figure 3:**
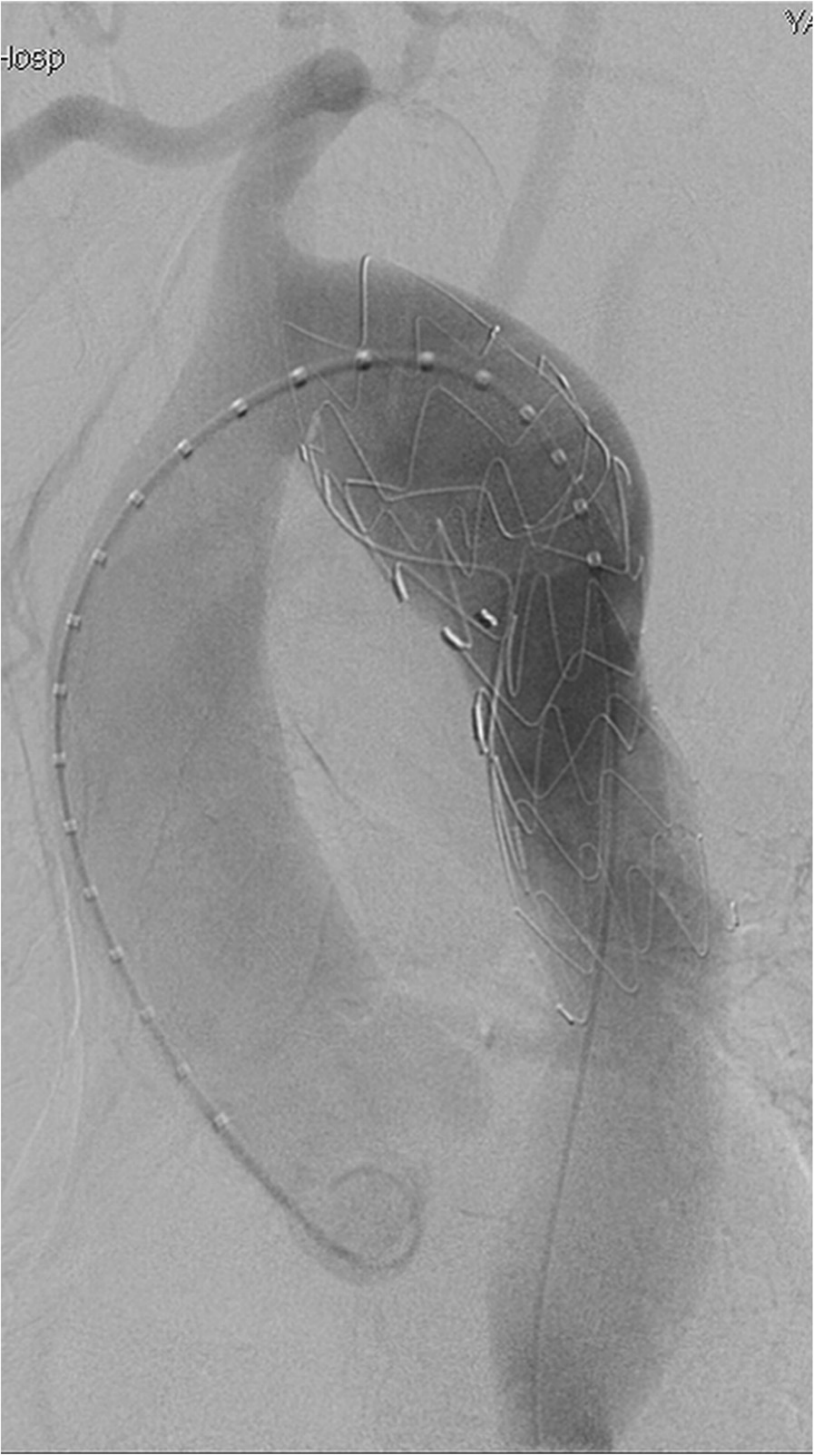
Postoperative angiogram after graft-stent implantation showed no evidence of endoleak, the false lumen of aortic dissection disappeared.

**Figure 4:**
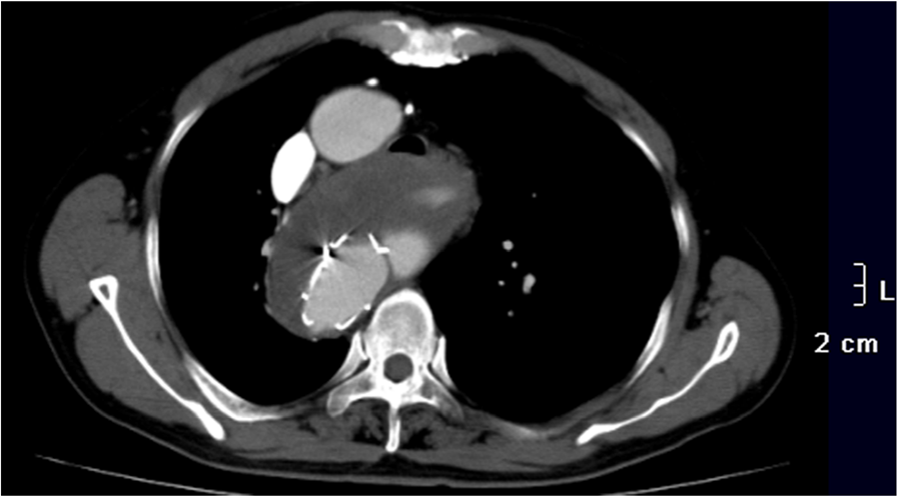
After 18 months follow-up, CTA documented that the false lumen was largely thrombosed only with a mild type II endoleak and a mild descending aortic expansion.

The procedure was performed under general endotracheal anesthesia and full hemodynamic monitoring one day later. The right femoral artery was exposed and cannulated with a 6-French catheter sheath, from which an angiographic mark catheter was put into the aortic true lumen via a 0.035 inch guidewire. Digital subtraction angiography (DSA) showed a right-sided aortic arch, type B aortic dissection, and dominanted right vertebral artery (Figure 2). We locked the “C” arm in right anterior oblique (RAO) 45° to unfold the aortic arch, and cannulated with a 21-French delivery introducer sheath via a 0.035 inch Lunderquist super stiff guidewire through the right femoral artery. A stent-graft (Talent, 4242150, Medtronic, USA) was accurately deployed. Completion angiogram revealed no evidence of type I endoleak, sealing of the false lumen, no visualization of left subclavian artery and no visceral vessels malperfused (Figure 3). His postoperative course was uneventful and discharged postoperative day 4. No complaint and complication was documented during 18 months follow-up. The CTA demonstrated that the false lumen was largely thrombosed only with a mild type II endoleak and a mild descending aortic expansion *(The size of descending aortic is 66mm)* (Figure 4).

## Discussion

Right-sided aortic arch occurs in approximately 0.1% of the population. There are three major types [1]. Type I, with mirror-imaged branching of the major arteries. Type II, with aberrant subclavian artery. The ordering of the aortic arch branches as left common carotid, right common carotid, right subclavian, and LSA arising from Kommerell’s diverticulum from proximal to distal. Type III, with isolation of the subclavian artery. Type I represent 59% of all right aortic arches, type II 39.5%, and type III 0.8%.

In 1994, Dake and colleages [3] first reported successful transluminal placement of endovascular stent-grafts for the treatment of aortic dissection, and showed good early results. This technique is less invasive than traditional surgery with acceptable morbidity and mortality rates. Endovascular treatment of right-sided aortic arch aneurysms is appealing because it avoids a thoracotomy or median sternotomy, and it obviates the need for circulatory arrest, which is commonly required with the conventional approach. Okada and associates [4] demonstrated the feasibility of endovascular repair in a 76-year-old man with an aneurysm that involved a right-sided aortic arch. Naoum and colleagues [5] described the successful surgical repair in a 58-year-old man of descending thoracic aortic aneurysm with a right-sided aortic arch by the combined use of a left carotid to subclavian artery bypass followed by endovascular stent-graft placement. Yamazaki [2] first reported a successful stent-graft treatment in a 56-year-old man suffered from type B aortic dissection involving a right aortic arch. In the present case, the anatomic features made the landing zone between the right subclavian artery and the thoracic aortic intimal tear long enough to accommodate a stent-graft without causing basal artery ischemia. *And let the “C” arm in right anterior oblique (RAO) 45° other than left anterior oblique (LAO) to unfold the aortic arch intraoperatively.* After 18 months follow-up, there is no dizzy, no swoon, no left upper extremity necrosis, no extremities myodynamia degrade, no nervous system complications, and no dysphagia stridor. However, a mild type II endoleak and a mild descending aortic expansion most probably come from the LSA back-flow. We are planning to followup of the type II endoleak and aortic expansion, and ligate or embolize the LSA when necessary.

## Conclusion

Although early results are promising, long-term clinical efficacy and safety have yet to be confirmed.

## Consent

Written informed consent was obtained from the patient for publication of this Case report and any accompanying images. A copy of the written consent is available for review by the Editor-in-Chief of this journal.

## Competing interests

The author declare that he has no competing interests.

## References

[B1] Cinà CS Arena GO Bruin G Clase CM Kommerell’s diverticulum and aneurysmal right-sided aortic arch: a case report and review of the literature J Vasc Surg 2000 32 1208 1214 10.1067/mva.2000.108012 11107094

[B2] Yamazaki I Imoto K Ichkawa Y Stent-graft treatment of type B aortic dissection involving the right aortic arch (case report) Jpn Circ J 2000 64 727 728 10.1253/jcj.64.727 10981862

[B3] Dake MD Miller MC Semba CP Mitchell RS Walker PJ Liddell RP Transluminal placement of endovascular stent-grafts for the treatment of descending thoracic aortic aneurysms N Engl J Med 1994 331 26 1729 1734 10.1056/NEJM199412293312601 7984192

[B4] Okada K Sueda T Orihashi K Watari M Naito A Endovascular stent-graft repair for thoracic aortic aneurysm associated with right-sided aortic arch J Thorac Cardiovasc Surg 2001 122 185 186 10.1067/mtc.2001.113019 11436056

[B5] Naoum JJ Parenti JL LeMaire SA Coselli JS Endovascular repair of a right-sided descending thoracic aortic aneurysm with a right-sided aortic arch and aberrant left subclavian artery Ann Thorac Surg 2008 85 1074 1076 10.1016/j.athoracsur.2007.09.032 18291202

